# Mitochondrial impairment in microglia amplifies NLRP3 inflammasome proinflammatory signaling in cell culture and animal models of Parkinson’s disease

**DOI:** 10.1038/s41531-017-0032-2

**Published:** 2017-10-17

**Authors:** Souvarish Sarkar, Emir Malovic, Dilshan S. Harishchandra, Shivani Ghaisas, Nikhil Panicker, Adhithiya Charli, Bharathi N. Palanisamy, Dharmin Rokad, Huajun Jin, Vellareddy Anantharam, Arthi Kanthasamy, Anumantha G. Kanthasamy

**Affiliations:** 1 Department of Biomedical Science, Iowa State University, Ames, IA 50011 USA; 2 Present Address: Perelman School of Medicine, Abramson Family Cancer Research Institute, University of Pennsylvania, 421 Curie Boulevard, 642 BRB II/III, Philadelphia, PA 19104 USA; 3 Present Address: Institute for Cell Engineering, The Johns Hopkins School of Medicine, 733 North Broadway, Baltimore, MD 21210 USA

## Abstract

The NLRP3 inflammasome signaling pathway is a major contributor to the neuroinflammatory process in the central nervous system. Oxidative stress and mitochondrial dysfunction are key pathophysiological processes of many chronic neurodegenerative diseases, including Parkinson’s disease (PD). However, the inter-relationship between mitochondrial defects and neuroinflammation is not well understood. In the present study, we show that impaired mitochondrial function can augment the NLRP3 inflammasome-driven proinflammatory cascade in microglia. Primary mouse microglia treated with the common inflammogen LPS increased NLRP3 and pro-IL-1β expression. Interestingly, exposure of LPS-primed microglial cells to the mitochondrial complex-I inhibitory pesticides rotenone and tebufenpyrad specifically potentiated the NLRP3 induction, ASC speck formation and pro-IL-1β processing to IL-1β in a dose-dependent manner, indicating that mitochondrial impairment heightened the NLRP3 inflammasome-mediated proinflammatory response in microglia. The neurotoxic pesticide-induced NLRP3 inflammasome activation was accompanied by bioenergetic defects and lysosomal dysfunction in microglia. Furthermore, the pesticides enhanced mitochondrial ROS generation in primary microglia, while amelioration of mitochondria-derived ROS by the mitochondria-targeted antioxidant mito-apocynin completely abolished IL-1β release, indicating mitochondrial ROS drives potentiation of the NLRP3 inflammasome in microglia. Exposure to conditioned media obtained from mitochondrial inhibitor-treated, LPS-primed microglial cells, but not unprimed cells, induced dopaminergic neurodegeneration in cultured primary mesencephalic and human dopaminergic neuronal cells (LUHMES). Notably, our in vivo results with chronic rotenone rodent models of PD further support the activation of proinflammatory NLRP3 inflammasome signaling due to mitochondrial dysfunction. Collectively, our results demonstrate that mitochondrial impairment in microglia can amplify NLRP3 inflammasome signaling, which augments the dopaminergic neurodegenerative process.

## Microglia: Powerhouse breakdown aggravates neurodegeneration

A team of American researchers demonstrate that disruption of mitochondria in microglia contributes to inflammation and neurodegeneration. Anumantha G. Kanthasamy at Iowa State University in Ames, IA and colleagues examined the effect of pesticides known to impair mitochondrial function on proinflammatory signaling pathways in microglia, the brain’s immune cells. They found that both rotenone and tebufenpyrad specifically stimulated the NLRP3 inflammasome, a multi-protein complex implicated in neuroinflammatory processes. The pesticide-treated microglia were able to cause more damage to neuronal cells than the untreated ones, indicating that mitochondrial dysfunction in microglia augments neurodegeneration. The authors also show that in rodents chronically exposed to rotenone, which causes many of the features of Parkinson’s disease (PD), the NLRP3 inflammasome is activated. These findings contribute to better understand the mechanisms driving chronic neuroinflammation in PD.

## Introduction

Parkinson’s disease (PD) is the most common neurodegenerative movement disorder affecting around 2% of the US population over age 60. Its incidence is expected to rise dramatically with the advancing median age of the population, worsening the substantial socioeconomic burden on patients, their families and society. The main pathological hallmark of this disease is degenerating dopaminergic (DAergic) neurons within the nigrostriatal tract that project from the substantia nigra (SN) to the striatum, resulting in severely depleted striatal DA that clinically manifests as a range of debilitating motor symptoms.^1,2^ The underlying mechanisms of the neuronal degeneration are not well understood, but mitochondrial dysfunction, chronic inflammation, and oxidative stress have been implicated in different animal models of PD.^3–5^ The role of inflammation in PD was first suggested in 1988 when major histocompatibility complex molecules were shown to be upregulated in PD patients.^6^ Furthermore, various proinflammatory factors like tumor necrosis factor α (TNF-α) and IL-1β were found to be upregulated in cerebrospinal fluid and different regions of the brain in PD patients.^7^ In various animal models of PD, including nigrostriatal lesions with 6-OHDA, MPTP, and rotenone, a selective loss of DAergic neurons is accompanied by chronic neuroinflammation,^8–12^ partly mediated by microglia, the resident immune cells in the brain.^5,13–15^ Since, depending on stimuli, microglia secrete both anti-inflammatory and pro-inflammatory factors, as well as growth factors, they are critical for regulating neuronal survival.^16,17^ Though various inflammogens have been shown to stimulate microglia-mediated neuroinflammatory processes, the precise mechanisms underlying neuroinflammation remain equivocal. Understanding the mechanism driving chronic brain inflammation may lead to a better understanding of PD pathogenesis and progression.

Mitochondrial dysfunction and perturbations in mitochondrial dynamics in DAergic neurons are well established causes of neuronal degeneration in PD.^18–24^ Despite this, the link between mitochondrial impairment in microglia per se and neuroinflammation is not well characterized. Recent studies have implicated inflammasome activation in inflammatory neurodegenerative disorders^25,26^ like Alzheimer’s disease (AD), multiple sclerosis, and traumatic brain injury (TBI).^25,27^ Inflammasomes are multiprotein oligomers mainly formed by ASC, caspase-1, and the inflammasome component (e.g., NLRP3, NLRP1, NLRC4, AIM2). Multiple inflammasomes have been identified in microglia, astrocytes and neurons. The major function of an inflammasome is to cleave pro-IL-1β to IL-1β, or to produce IL-18 to enhance and sustain inflammation. Both NLRP3 and NLRP1 inflammasomes have been implicated in AD pathogenesis.^28,29^ NLRP3 inflammasome activation normally requires two signals for its function. Signal 1 activates the NFκB pathway facilitating pro-IL-1β and NLRP3 transcription and translation. Signal 2 forms the inflammasome complex comprising NLRP3, ASC, and caspase-1, which in turn cleaves pro-IL-1β to IL-1β. Signal 2 can vary from pathogens to aggregated proteins to ATP. Mechanisms underlying inflammasome complex formation remain unresolved.^30^

The current study addresses the putative link between mitochondrial impairment and inflammasome activation in microglial cells, which may help identify a mechanism behind chronic inflammation-driven neurodegeneration. Recently, we demonstrated in a DAergic neuronal cell culture model that mitochondria-impairing pesticides compromise mitochondrial dynamics (structure and function) by inhibiting mitochondrial complex-I.^21^ Here, we demonstrate that impairing mitochondrial function in primary microglial cells, which had been treated with the classical PD mitochondrial neurotoxicant rotenone and the acaricide tebufenpyrad, may play an important role in inducing the NLRP3 inflammasome. Furthermore, we demonstrate activation of the NLRP3 inflammasome in a chronic rotenone animal model of PD. We also demonstrate that mitochondrially derived ROS contributes to inflammasome activation by utilizing a mitochondrially targeted derivative of apocynin.

## Results

### Rotenone and tebufenpyrad activate NLRP3 inflammasome in primary microglia

**Fig. 1:**
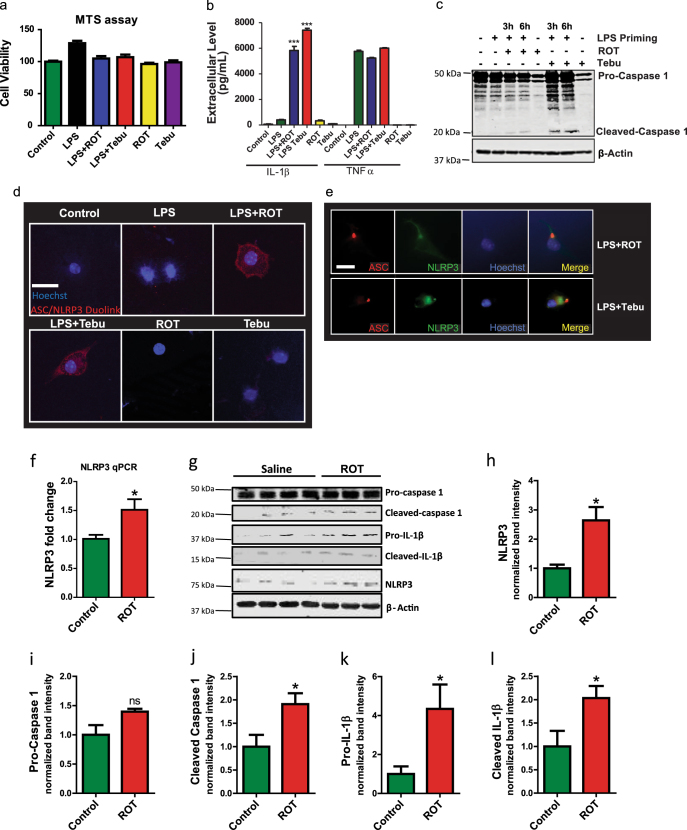
Rotenone and tebufenpyrad activate the NLRP3 inflammasome in primary murine microglia. **a** MTS assay of cell viability after treating lipopolysaccharide (LPS)-primed primary microglia with the mitochondrial complex-1 inhibiting pesticides rotenone (ROT) or tebufenpyrad (Tebu) (both 1 µM) for 24 h. **b** Luminex assay showing an increased IL-1β release from LPS-primed microglia after pesticide treatments for 24 h. Treatment with rotenone or tebufenpyrad did not alter TNFα release. **c** Western blot analysis of pesticide-treated, primed microglial cells showing the cleavage of pro-caspase-1 to its active form caspase-1 p20. **d** Duolink proximal ligation assay reveals ASC and NLRP3 interaction in pesticide-exposed, primed microglial cells but not in unprimed cells following 3 h of pesticide exposure. Scale bar, 15 μm. **e** ICC showing ASC speck formation in pesticide-treated primed microglial cells for 2 h. Scale bar, 20 μm. **f** q-RT-PCR analysis of vehicle- and rotenone-gavaged (*n* = 4 each group, 30 mg/kg for 28 days) mice for striatal NLRP3 gene expression. **g** Western blot analysis for vehicle- and rotenone-gavaged (30 mg/kg for 28 days) mice for NLRP3, caspase-1 and IL-1β. (H-K) Densitometric analysis of Western blot for NLRP3. **h**, caspase-1 **i**, caspase-1 p20 **j**, pro-IL-1β **k**, and cleaved IL-1β **l**. For all Western blots, samples derive from the same experiment and were processed in parallel. Data analyzed via Student’s *t* test, or via two-way ANOVA with Bonferroni adjustment, *p < 0.05, **p < 0.01, ***p < 0.001 and are represented as Mean ± SEM with *n* = 3–8

We utilized two mitochondrial complex-1 inhibitors, rotenone and tebufenpyrad, to confirm cross-talk between mitochondrial dysfunction and microglial NLRP3 inflammasome signaling. Exposure of lipopolysaccharide (LPS)-primed (1 µg/mL for 3 h) or unprimed primary mouse microglia cells to a concentration (1 μM for 24 h) of rotenone or tebufenpyrad sufficient to inhibit mitochondrial complex-1^31^ did not induce cell death, as measured by MTS assay (Fig. 1a). Next, Luminex multiplex cytokine assays revealed a dramatic increase in the levels of IL-1β, but not that of TNF-α, confirming that the rotenone and tebufenpyrad treatments of primed cells induced release of the pro-inflammatory cytokine that is mediated by inflammasome signaling (Fig. 1b). Pesticide treatments alone did not increase either IL-1β or TNF-α in unprimed primary microglia (Fig. 1b).

Next, LPS-primed or unprimed primary microglia were exposed to rotenone and tebufenpyrad for 3 and 6 h. Western blotting (Fig. 1c) revealed a time-dependent increase in cleaved caspase-1 levels when compared to LPS-primed and unprimed microglia, although cleaved caspase-1 did increase slightly in LPS-primed microglia. Both the enhanced release of IL-1β and cleavage of caspase-1 suggest the induction and activation of inflammasome signaling following mitochondrial complex-1 inhibition. Next, ICC analysis revealed that NLRP3 immunoreactivity is indeed induced in rotenone- and tebufenpyrad-treated, LPS-primed microglia, but not in control microglia (Supplementary Fig. 1). As expected, NLRP3 induction also occurred in the LPS-priming alone group. Exposure of LPS-primed microglia to a secondary stimulus like rotenone or tebufenpyrad after NLRP3 induction resulted in the association of NLPR3 with ASC,^32^ as revealed by the proximal ligation Duolink assay (Fig. 1d). Furthermore, both rotenone-treated and tebufenpyrad-treated, primed microglial exhibited inflammasome activation-linked ASC speck formation^33^ (Fig. 1e). These data collectively support the two-pronged approach of inflammasome activation as previously described.^27,32^ Signal 1, which in our study is LPS, induces the expression of NLRP3 and pro-IL-1β, but not the cleavage of pro-IL-1β to its active form IL-1β. Signal 2, which in our study are the mitochondrial inhibitors, leads to formation of the inflammasome complex consisting of NLRP3, ASC and caspase-1. This complex formation leads to activation of caspase-1, which in-turn cleaves pro-IL-1β to IL-1β. That is why we observed increased IL-1β release only in the LPS + Rot and LPS + Tebu groups.

Wilson et al.^34^ have shown that NLRP3 inflammasome activation also induces IL-18 release along with IL-1β. Thus, we treated LPS-primed mouse microglia with either tebufenpyrad or rotenone for 2 h and then measured pro-IL-18 by qRT-PCR mRNA analysis. In line with previous reports,^32^ both pro-IL-1β mRNA and pro-IL-18 levels increased significantly in LPS-primed and pesticide treatment groups (Supplementary Fig. 2A–B).

A study by Sherer et al.^35^ reported that the IC50 for complex I inhibition is in nanomolar range for both rotenone and tebufenpyrad. The cell type used in their study is of neuronal lineage, which is more susceptible to complex I inhibitors than macrophage-like cells, including microglial cells used in our study. A much higher dose of rotenone (10 µM) has been previously used in inflammasome studies.^32^ High micro-molar doses of rotenone are known to induce acetylation of tubulin.^36^ Microtubule acetylation has also recently been linked to NLRP3 inflammasome activation.^37^ Misawa et al. ^37^ reported that SIRT2 downregulation leads to acetylation of α-tubulin, which modulates NLRP3 inflammasome activation. Hence, to confirm if our micro-molar dose of pesticides affects microtubule assembly and inflammasome activation, primary microglial cells were treated with 1 µM rotenone and tebufenpyrad for 3 h following LPS priming. ICC analysis revealed that SIRT2 and acetylated α-tubulin levels did not change significantly following pesticide exposure (Supplementary Fig 3). These data suggest that pesticide-induced inflammasome activation is not dependent on microtubule assembly at early time points.

Next, we utilized a neuron-microglia co-culture system to see if pesticide exposure alone can prime the microglial cells. Mouse MN9D dopaminergic neuronal cells were grown on inserts placed in wells growing microglial cells. The cells were treated with 1 µM rotenone for 6 h, after which q-RT-PCR analysis revealed that pesticide exposure induced NLRP3 (Supplementary Fig. 4A) and pro-IL-β (Supplementary Fig. 4B) in the co-cultured microglial cells. To further validate our in vitro study in vivo, we made use of the rotenone mouse model of PD.^38^ Mice were gavaged daily for 28 days with 30 mg/kg rotenone. Rotenone significantly increased NLRP3 gene expression as revealed by qPCR analysis of striatal lysates (Fig. 1f). Immunoblot analysis showed that rotenone also significantly increased inflammasome markers such as NLRP3, cleaved caspase-1 p20, pro-IL-1β and cleaved IL-1β (Figs. 1g–l).

Next, we also utilized the rat model of rotenone. Rats were injected with 2.8 mg of rotenone for 4 days and sacrificed after 3 months.^11^ Our q-RT-PCR analysis revealed that, like in the mouse model, rotenone induced NLRP3 expression in rats (Supplementary Fig. 5A). Moreover, immunohistochemical analysis revealed that rotenone exposure upregulated NLRP3 in the IBA1-positive microglial cells (Supplementary Fig. 5B). We wanted to clarify that the LPS priming used in the in vitro model acts as signal 1 to activate the NFκB pathway. However, in the in vivo models, LPS priming is not necessary since the rotenone-induced neuronal stress can itself act as signal 1.^30^ Furthermore, including the LPS priming step in vivo will make the model more complex as LPS can induce inflammasome activation.^39^ Also, unlike an LPS priming step for in vitro models, it is not possible to get rid of the LPS in vivo after a certain time. These results collectively showed that mitochondrial complex-1 inhibition can lead to NLRP3 inflammasome activation in an environmental pesticide toxicant-driven mouse model of PD. Together, these findings indicate that rotenone and tebufenpyrad can activate the NLRP3 inflammasome in primary mouse microglia.

### Mitochondrial complex-1 inhibition induces a dose-dependent release of IL-1β in primed microglia through the NLRP3 inflammasome pathway

**Fig. 2:**
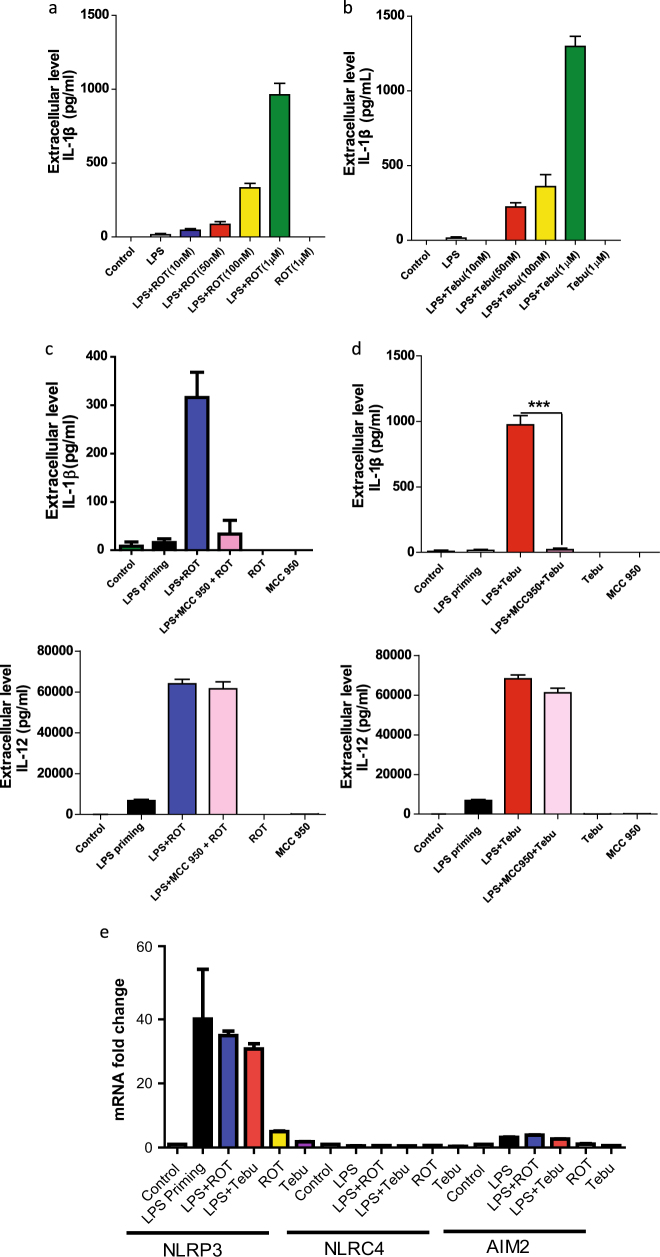
Rotenone and tebufenpyrad induce a dose-dependent release of IL-1β in primed microglial cells through the NLRP3 inflammasome pathway. **a**–**b** Luminex assay showing a dose-dependent increase in IL-1β release after treating primed primary microglial cells for 24 h with rotenone **a** and tebufenpyrad **b**. **c** Luminex assay showing inhibition of IL-1β release after co-treating primed microglial cells with rotenone and MCC-950, and showing no change in IL-12 release. **d** Luminex assay showing inhibition of IL-1β release after co-treating primed microglial cells with MCC-950 and tebufenpyrad, and showing no change in IL-12 release. **e** q-RT-PCR analysis showing different inflammasome gene expression after treatment of LPS-primed primary mouse microglia with 1 µM rotenone and tebufenpyrad for 2 h. Data analyzed via two-way ANOVA with Bonferroni adjustment, *p < 0.05, **p < 0.01, ***p < 0.001 and are represented as Mean ± SEM with *n* = 3–8

To test if the effects of rotenone and tebufenpyrad on inflammasome activation are dose-dependent, we exposed LPS-primed primary mouse microglia to 10–1000 nM of rotenone or 50–1000 nM of tebufenpyrad for 24 h and then the medium was used for determining the levels of extracellularly released IL-1β. Luminex assays revealed both rotenone (Fig. 2a) and tebufenpyrad (Fig. 2b) induced a dose-dependent release of IL-1β from LPS-primed cells. Indeed, even doses as low as 50 nM induced NLRP3 inflammasome-mediated IL-1β release.

To further confirm whether the rotenone-induced and tebufenpyrad-induced IL-1β release is due to activation of the NLRP3 inflammasome, LPS-primed and unprimed primary microglial cells were pretreated with 100 nM MCC-950, a potent NLRP3 inflammasome inhibitor,^40^ for 1 h followed by co-treatment with rotenone or tebufenpyrad for 6 h. An MTS assay revealed that 100 nM MCC-950, tested alone or in combination with the pesticides, did not lead to cell death, confirming that the NLRP3 inhibitor’s effect was not due to cytotoxicity (Supplementary Fig. 6). Also, the MCC-950 pre-treatment almost completely blocked the rotenone-induced secretion of IL-1β (Fig. 2c) and that of tebufenpyrad (Fig. 2d). MCC-950 does not block the rotenone-induced or tebufenpyrad-induced release of IL-12, which is not dependent on NLRP3 activation (Fig. 2c–d, right panels).

The other NLRP family members AIM2 and NLRC4 can also mediate the release of IL-1β.^41^ Thus, we treated LPS-primed mouse primary microglia with the mitochondrial inhibitors for 2 h and then measured various inflammasomes by qRT-PCR mRNA analysis. We observed a huge induction in NLRP3 mRNA levels only in LPS-primed microglia without changes in AIM2 or NLRC4 levels (Fig. 2e), again suggesting specificity towards NLRP3 induction in primary microglia. Together, these results strongly suggest that the complex-1 inhibitor-induced release of IL-1β is indeed mediated by NLRP3 activation.

### Mitochondrial complex-1 inhibitors induce structural and functional changes in microglia

**Fig. 3:**
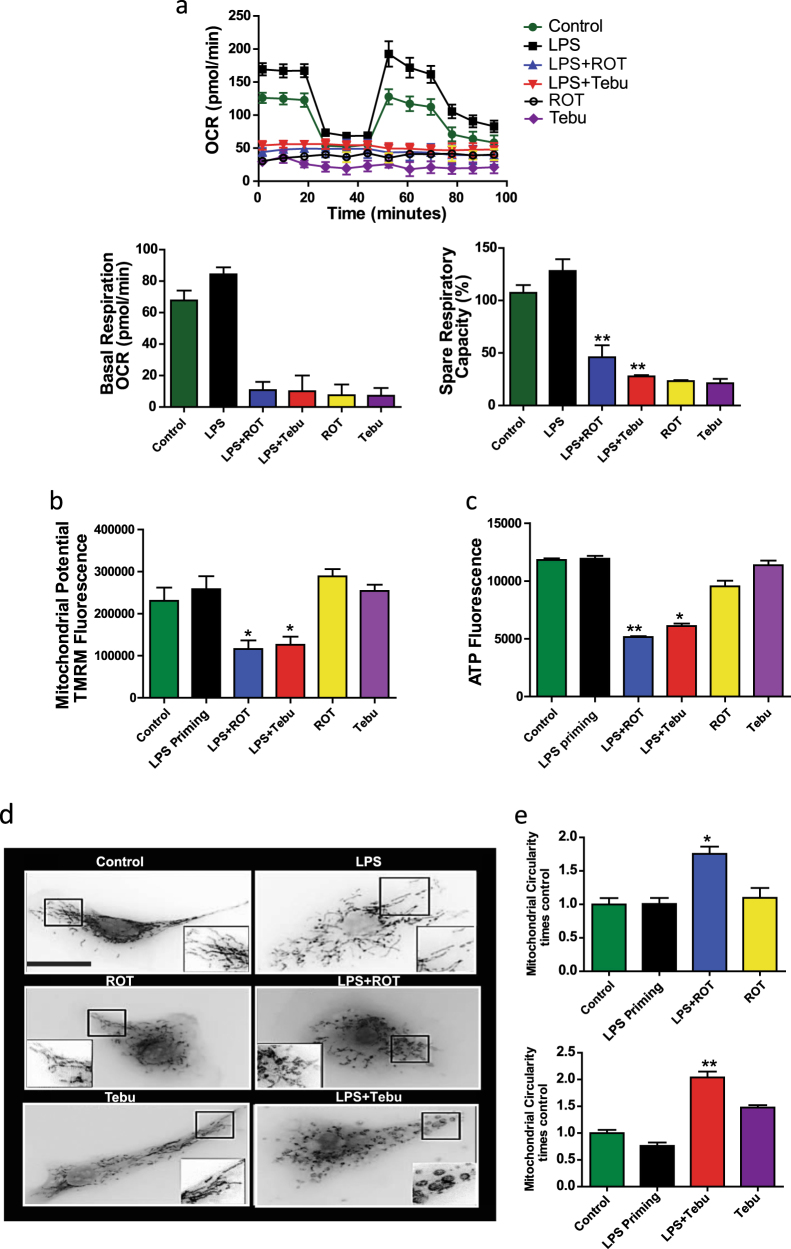
NLRP3 inflammasome activation is accompanied by mitochondrial functional and structural damage. **a** Seahorse Mito-Stress assays showing impaired mitochondrial bioenergetics in LPS-primed primary microglial cells treated with pesticides. **b** Quantification of TMRM fluorescence staining showing mitochondrial potential in untreated and treated primary microglia. **c** ATP assays in LPS-primed mouse primary microglia treated with rotenone and tebufenpyrad. **d** MitoTracker assays show changes in mitochondrial morphology in LPS-primed primary microglia treated with pesticides. Scale bar, 15 μm. **e** Circularity, indicative of mitochondrial fragmentation, increased after pesticide treatment. Data analyzed via two-way ANOVA with Bonferroni adjustment, *p < 0.05, **p < 0.01, ***p < 0.001 and are represented as Mean ± SEM with *n* = 3–8

Next, we characterized the microglial structural and functional changes underlying cross-talk between mitochondrial dysfunction and NLRP3 inflammasome activation. We first treated LPS-primed primary microglial cells with 1 µM rotenone or tebufenpyrad for 3 h. Exposure of LPS-primed cells to both complex-1 inhibitors impaired mitochondrial bioenergetics as indicated by compromised basal respiration, ATP-linked respiration, and spare respiratory capacity, which were measured using a Seahorse Bioscience XFe24 analyzer (Fig. 3a). Next, we treated LPS-primed primary microglia for 2 h with rotenone or tebufenpyrad and then stained them with TMRM, a dye which is readily sequestered into active mitochondria and stains them according to their potential. Pesticide-treated primed microglia exhibited reduced TMRM fluorescence (Fig. 3b) compared to unprimed cells, suggesting inflammasome-related mitochondrial dysfunction. Furthermore, an ATP assay (Fig. 3c) revealed that the pesticide-treated, primed microglia produced less ATP when compared to unprimed cells, suggesting that their mitochondria were functionally damaged at an early stage of inflammasome activation. We tested for additional markers of pesticide-induced mitochondrial structural damage in primed primary microglial cells treated with 1 µM rotenone or tebufenpyrad by staining them for 6 h with MitoTracker Red. Quantification of fluorescence revealed that mitochondria in pesticide-treated, LPS-primed microglial cells exhibited greater fission-related circularity and solidity than did non-treated cells (Fig. 3d-e).

To identify if the mitochondrial dysfunction is an effect of inflammasome activation, we co-treated primary microglial cells with the NLRP3-specific inhibitor MCC-950 (100 nM) and 1 µM rotenone or tebufenpyrad for 3 h. The mitochondrial stress assay revealed that inhibiting the NLRP3 inflammasome did not reverse the mitochondrial defects (Supplementary Fig. 7a–b). These data indicate the probable role of mitochondria upstream of inflammasome activation.

Together with inflammasome results, these findings indicate that complex-1 inhibition-associated NLRP3 inflammasome activation is accompanied by mitochondrial structural and functional dysfunction in microglial cells.

### Mitochondrial superoxide generation plays a role in rotenone-induced and tebufenpyrad-induced NLRP3 inflammasome activation

**Fig. 4:**
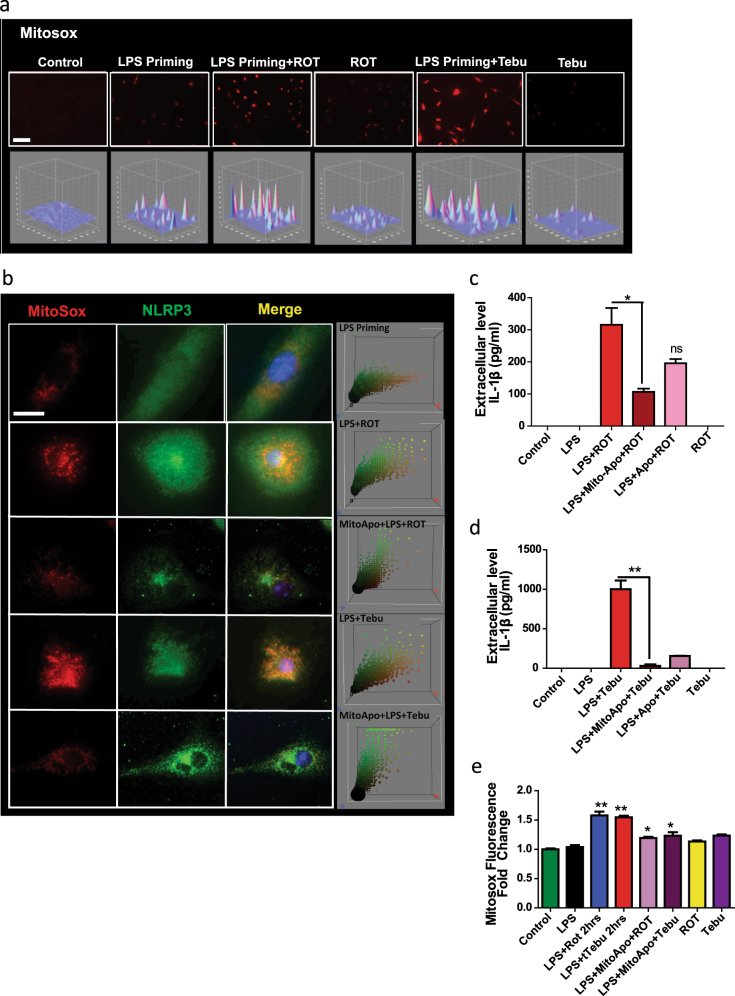
Mitochondrial superoxide generation plays a role in pesticide-induced NLRP3 inflammasome activation. **a** ICC showing MitoSox generation from pesticide-treated primed microglial cells. Lower panel shows interactive 3D surface plot from corresponding ICC, which shows the intensity of superoxide generation. Scale bar, 100 μm. **b** ICC showing co-localization of mitochondrial superoxide generation and NLRP3. The right-most panel is the representative 3D color plot. Mito-apocynin reduced this co-localization between NLRP3 and MitoSox. Scale bar, 15 μm. **c**–**d** Luminex assays demonstrate that mito-apocynin reduced IL-1β secretion from primed microglial cells treated with rotenone **c** and tebufenpyrad **d**. **e** MitoSox assays reveal that mito-apocynin reduced pesticide-induced superoxide generation in pesticide-treated primed microglial cells. Data analyzed via two-way ANOVA with Bonferroni adjustment, *p < 0.05, **p < 0.01, ***p < 0.001 and are represented as Mean ± SEM with *n* = 3–8

Damaged mitochondria release superoxide (O2-), which not only causes oxidative damage to cells,^42^ but also activates inflammasome signaling in peripheral immune cells.^43,44^ To decipher the role of superoxide in complex-1 inhibition-associated NLRP3 inflammasome activation, we treated primed microglia with rotenone or tebufenpyrad for 3 h and then stained with MitoSox dye to detect mitochondrial superoxide generation. The pesticide-treated primed microglia exhibited intense MitoSox staining, but unprimed and primed-only cells did not (Fig. 4a). Next, real-time images taken every 1 h of primed wild-type microglial cells treated with tebufenpyrad or rotenone revealed intense MitoSox fluorescence in the pesticide-treated groups within 2 h (Supplementary Video. 1–3).

Previous studies by Dr. Jorg Tschopp^32^ and others^45^ have shown that blocking mitophagy increases superoxide generation, which leads to NLRP3 inflammasome activation. Furthermore, upon activation, NLRP3 redistributes and colocalizes with the mitochondria. Mitochondrial prion-like proteins called MAVS mediate this redistribution of NLRP3 to mitochondria.^45^ Since NLRP3 has been shown to translocate to mitochondria upon inflammasome activation,^32^ we next examined whether NLRP3 co-localizes with superoxide generated by damaged mitochondria. LPS-primed or unprimed primary mouse microglia treated with rotenone or tebufenpyrad for 3 and 6 h were stained for MitoSox and NLRP3. A marked increase in NLRP3 immunoreactivity overlapping with the increased MitoSox Red fluorescence was observed in pesticide-treated cells (Fig. 4b), suggesting that the NLRP3 mitochondrial translocation depends on mitochondrial superoxide generated from damaged mitochondria. The degree of co-localization was further visualized as a shift in the 3D color plot (Fiji plugin), signifying that MitoSox and NLRP3 overlap, merging along the diagonal on the red and green channel axes.

We also hypothesized that reducing mitochondrial superoxide levels would decrease inflammasome activation and in turn lower IL-1β release. For this study, we pretreated primary microglia for 1 h with 40 µM of mito-apocynin (Mito-Apo), a mitochondria-targeted derivative of apocynin that was previously shown by our group to reduce oxidative stress and superoxide generation.^9,46^ This was followed by a 3h rotenone or tebufenpyrad treatment of primed microglia. Mito-Apo pretreatment significantly reduced superoxide generation as evidenced by reduced MitoSox staining (Fig. 4e). Furthermore, Luminex assays revealed that Mito-Apo significantly reduced rotenone- and tebufenpyrad-induced IL-1β release in primed cells (Fig. 4c-d). Mito-Apo was more effective in reducing inflammasome activation than its precursor, apocynin.

Interestingly, ICC analysis further revealed that Mito-Apo not only reduced the superoxide generation, but also reduced the NLRP3 colocalization, with the superoxide-generating mitochondria indicating the probable upstream role of mitochondria in NLRP3 inflammasome activation (Fig. 4b). These findings suggest that inhibition of mitochondrial superoxide generation can reduce NLRP3 inflammasome activation and that the mitochondrial ROS plays a central role in the functional interaction between mitochondrial dysfunction and NLRP3 inflammasome activation.

### Lysosomal dysfunction enhances rotenone- and tebufenpyrad-induced NLRP3 inflammasome activation in microglia

**Fig. 5:**
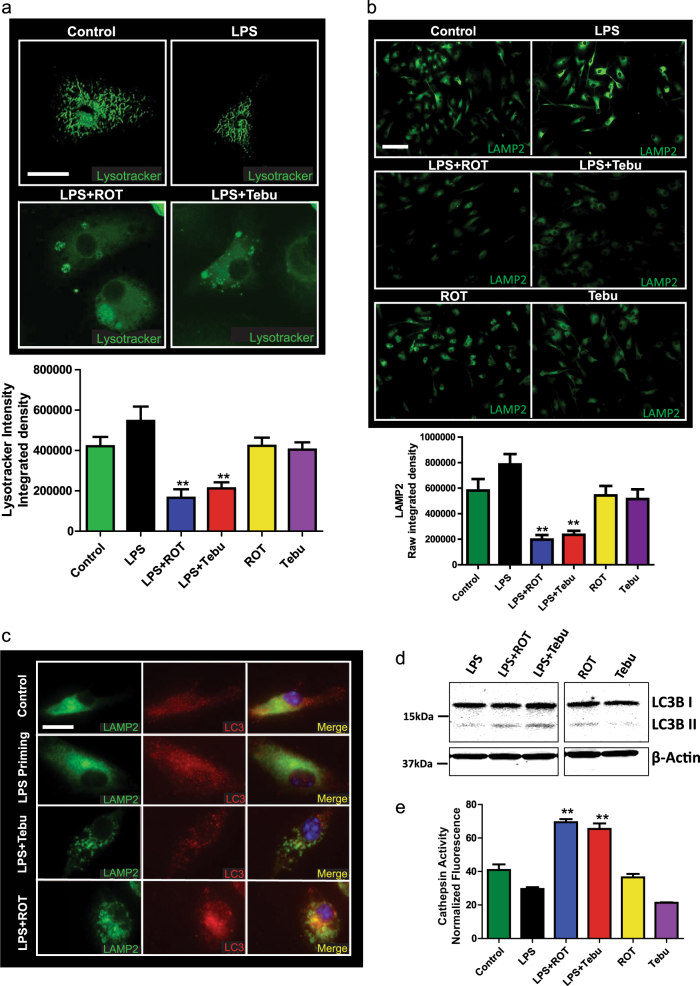
Lysosomal dysfunction enhances pesticide-induced NLRP3 inflammasome activation in microglia. **a** Confocal microscopy showing a lower LysoTracker fluorescence intensity in primed microglial cells treated with pesticides (1 µM). Scale bar, 15 μm. **b** ICC for LAMP2 staining showing a lower LAMP2 immunoreactivity in primed microglial cells treated with pesticides, implying lysosomal damage. Scale bar, 100 μm. **c** 60× imaging for LAMP2 ICC showing structural changes in lysosomes in primed microglial cells treated with pesticides. Scale bar, 20 μm. **d** Western blot analysis reveals increased LC3-II in primed microglial cells exposed to 1 µM pesticides for 3 h. Samples derive from the same experiment and gels/blots were processed in parallel. **e** Cathepsin activity assay revealed that 1 µM rotenone or tebufenpyrad for 6 h induced higher activity of this enzyme in primed microglial cells. Data analyzed via two-way ANOVA with Bonferroni adjustment, *p < 0.05, **p < 0.01, ***p < 0.001 and are represented as Mean ± SEM with *n* = 3–8

Healthy cells degrade damaged mitochondria through a lysosomal mechanism called mitophagy.^47^ Since blocking mitophagy has already been shown to induce inflammasome activation,^32^ we hypothesized that lysosomal dysfunction may add to the accumulation of damaged mitochondria and more superoxide. We treated primary microglia with rotenone or tebufenpyrad for 6 h following LPS priming. Staining with LysoTracker dye revealed markedly reduced LysoTracker fluorescence in pesticide-treated primed cells relative to unprimed cells, indicating the occurrence of lysosomal dysfunction in pesticide-treated primed microglial cells (Fig. 5a).

Next, to determine the extent of lysosomal damage, we stained the primary cells with LAMP2, a lysosomal marker, and found a significant reduction of LAMP2 immunoreactivity in rotenone-treated and tebufenpyrad-treated groups (Fig. 5b). High magnification imaging of primary microglia double-stained with LAMP2 and the autophagy marker LC3 revealed that LAMP2 forms large vesicle-like structures in response to tebufenpyrad and rotenone (Fig. 5c). Western blot analysis further revealed that 1 µM pesticides induced LC3-II in primed microglial cells after 3 h (Fig. 5d). Lysosomal membrane rupture leads to cathepsin release and activation, which have been shown to further modulate NLRP3 inflammasome activation.^48^ Since our ICC analysis showed reduced LAMP2, we performed a cathepsin D assay (Fig. 5e). This assay revealed that 1 µM rotenone or tebufenpyrad induced the activity of this lysosomal enzyme, further indicating lysosomal dysfunction in microglia. Collectively, these findings indicate that the loss of lysosomal activity may lead to the accumulation of damaged mitochondria, which can result in the overproduction of superoxide leading to enhanced, persistent inflammasome activation.

### Conditioned medium from complex-1 inhibitor-treated primed microglia induce DAergic neurodegeneration in primary mesencephalic cultures and human DAergic cells

**Fig. 6:**
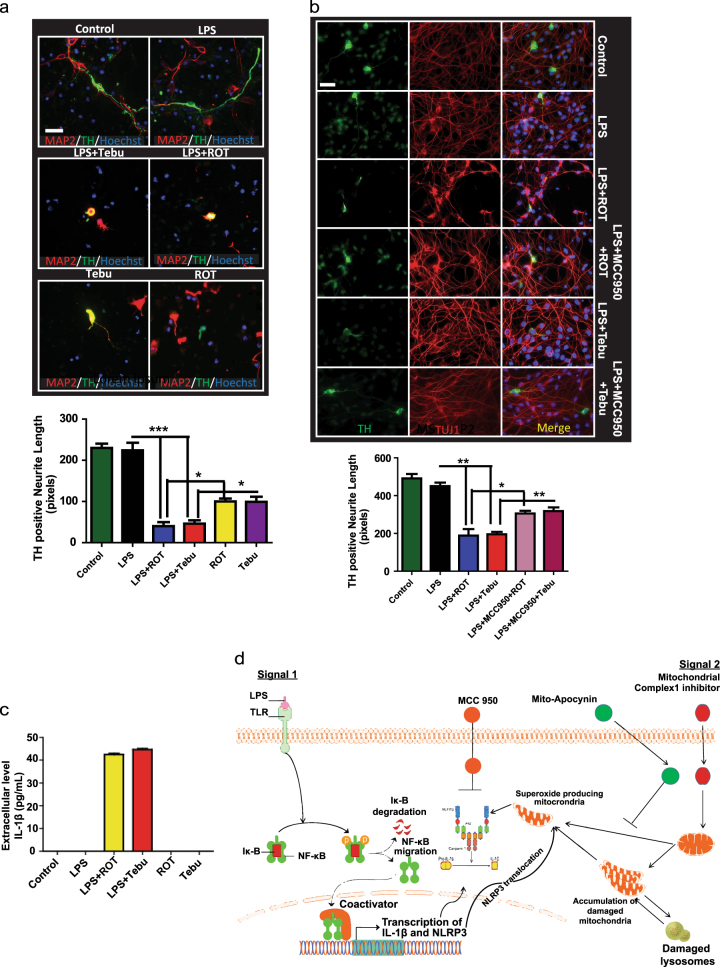
Conditioned medium from primary microglia treated with mitochondrial complex-1 inhibitors leads to DAergic neuronal toxicity. **a** ICC analysis of TH^+^ neurons treated with conditioned medium from primed microglial cells treated with 1 µM rotenone or tebufenpyrad for 24 h. The length of TH^+^ neurites was determined using ImageJ. Conditioned medium from primed-microglial cells treated with tebufenpyrad reduced TH^+^ neurite length. Scale bar, 100 μm. **b** ICC analysis of differentiated LUHMES cells treated with conditioned medium from primed microglial cells treated with 1 µM tebufenpyrad or rotenone for 24 h and cotreated with MCC-950. Scale bar, 100 μm. **c** Luminex analysis of the conditioned medium used to treat LUHMES cells. **d** A schematic representation illustrating the signaling cascade involved in pesticide-induced NLRP3 inflammasome activation in microglial cells; drawing created by S. Sarkar using biomedical PowerPoint toolkit from Motifolio. Data analyzed via two-way ANOVA with Bonferroni adjustment, *p < 0.05, **p < 0.01, ***p < 0.001 and are represented as Mean ± SEM with *n* = 3–8

To investigate the functional importance of complex-1 inhibition-induced microglial NLRP3 inflammasome activation, we used both mouse primary neuronal culture and differentiated LUHMES cells and conditioned medium from complex-1 inhibitor-treated primary microglial cells. Media collected from primed and unprimed microglial cells treated with tebufenpyrad or rotenone for 6 h was used to treat mouse primary nigral neurons to induce neuronal damage. TH-ICC analysis revealed significantly reduced neurite length of TH-positive neurons treated with medium from both rotenone and tebufenpyrad-treated primed cells, but not from unprimed cells (Fig. 6a). Interestingly, TH-negative neurite length did not significantly decrease, suggesting that these pesticides are specifically toxic to TH-positive neurons (Supplementary Fig. 8). This evidence suggests that complex-1 inhibition-triggered NLRP3 inflammasome activation in microglia is capable of inducing DAergic neuronal loss. The conditioned medium from the unprimed cells exposed to pesticides also caused neuronal damage. Hence, to further determine that the DAergic neuronal injury results from pesticide-induced inflammasome activation and not the pesticides present in the conditioned medium, we washed the LPS-primed cells to completely remove all the pesticides following a 6-h pesticide treatment and then kept the cells in 2% fresh medium for 18 h. Cells were also co-treated with MCC-950 (100 nM) during the 6-h pesticide exposure. When the pesticide-free medium was used to treat LUHMES cells, only cells incubated with conditioned medium from pesticide-treated, LPS-primed microglia exhibited a significant loss of TH^+^ cells (Fig. 6b). Conditioned medium co-treated with MCC-950 protected against the loss of neurite length (Fig. 6b). A Luminex bioassay of the cytokine profile further revealed that the conditioned medium collected from pesticide-treated, primed microglial cells, but not from the LPS-priming alone cells, contained IL-1β (Fig. 6c). These results further support the hypothesis that inflammasome activation resulting from mitochondrial dysfunction in microglia contributes to DAergic degeneration.

## Discussion

Recent studies suggest that the NLRP3 inflammasome plays an important role in mediating neuroinflammation;^41^ however, the key upstream signaling mechanisms that govern inflammasome activation have yet to be elucidated. Despite ample evidence that mitochondrial dysfunction is a key player in neurodegeneration, the link between mitochondrial dysfunction and neuroinflammation is much weaker. Our study bridges that gap in the field with a major focus on understanding the role of mitochondrial complex-1 inhibition in microglial cells and its relevance to inflammation, more specifically to NLRP3 inflammasome signaling. Here, we demonstrate that the complex-1 inhibitors rotenone and tebufenpyrad induce NLRP3 inflammasome activation in primary microglial cells by modulating mitochondrial dynamics in microglia itself. We also show that mitochondrial superoxide generation in microglia plays an important role in NLRP3 inflammasome activation. Furthermore, mito-apocynin, a derivative of mitochondria-targeted apocynin, attenuates inflammasome activation by decreasing superoxide generation. Finally, we demonstrate activation of the NLRP3 inflammasome in a chronic rotenone mouse model of PD.

A recent study by Zhou et al.^32^ has linked NLRP3 inflammasome activation to mitochondrial dysfunction in macrophages. The authors reported that upon activation, the NLRP3 complex migrates to the mitochondria, though its role in mitochondria is not well understood. In our present study, we show that the potent complex-1 inhibitors rotenone and tebufenpyrad both induce NLRP3 inflammasome activation in LPS-primed primary mouse microglia. As early as 3 h, both rotenone and tebufenpyrad induced a proteolytic conversion of pro-caspase-1 to active caspase-1 in LPS-primed primary microglia (Fig. 1), the first step in inflammasome activation. Rotenone and tebufenpyrad also induced the next step in the inflammasome activation process, namely caspase-1-mediated conversion of pro-IL-1β to IL-1β. Tebufenpyrad is more potent than rotenone at the same dose and leads to more caspase-1 p20 production and IL-1β secretion (Fig. 1b-c). ASC, the adapter protein for the NLRP3 inflammasome, forms spec-like structures and propagates in a “prionoid” fashion to propagate the inflammasome.^33,49^ As expected, both rotenone and tebufenpyrad treatments led to the formation of ASC specs (Fig. 1e). Furthermore, our results indicate that these complex-1 inhibitors induced the release of IL-1β in a dose-dependent manner, beginning at an extremely low dose of 50 nM (Fig. 2). To determine which inflammasome is mediating the generation and release of IL-1β, we used the NLRP3-specific inhibitor MCC-950.^40^ MCC-950 completely abolished the rotenone-induced and tebufenpyrad-induced release of IL-1β from LPS-primed microglia, thus confirming that NLRP3 is the major inflammasome activated by complex-1 inhibition. In terms of understanding the role of these environmental toxicants in sustained inflammation, our study demonstrates that pesticides can activate the NLRP3 inflammasome in primary microglial cells, thereby contributing to chronic inflammation.

Recent studies have focused on the role of mitochondria in NLRP3 inflammasome activation^50,51^ in different immune cells like bone marrow-derived macrophages. Shimada et al.^52^ revealed that mitochondrial DNA can activate the NLRP3 inflammasome. Inhibition of mitophagy has been shown to induce NLRP3 inflammasome activation. Furthermore, upon activation, NLRP3 translocates to the mitochondria. Recent studies have further shown that during activation the mitochondrial adaptor MAVS, through a prion-like mechanism, facilitates this translocation and activation of the NLRP3 inflammasome.^45^ Other studies revealed that mitochondrially generated reactive oxygen species (mROS) can activate macrophagic NLRP3 inflammasomes in response to alum or nigericin.^50,53^ Mitochondrial dysfunction and mROS are druggable targets for reducing inflammasome activation.^54^Apart from mounting evidence that mitochondrial dysfunction is a major regulatory signal for inflammasome activation in non-neuronal systems,^32,50,51,54,55^ our study further strengthens the role of mitochondrial impairment in NLRP3 inflammasome activation in microglial cells. We show that NLRP3 inflammasome activation is associated with altered mitochondrial dynamics, including functional and structural damage, as well as mitochondrial membrane potential changes (Fig. 3). Also, mitochondrial capacity for ATP generation was diminished when LPS-primed microglia were treated with complex-1-inhibiting pesticides. After prolonged exposure, structural damage to the mitochondria was manifested as increased mitochondrial circularity. Inhibiting inflammasome activation did not alter the mitochondrial dysfunction induced by the pesticides, indicating that the mitochondrial damage may be upstream of inflammasome activation. Superoxide generation preceded discernible mitochondrial damage,^32^ and interestingly, our studies show that NLRP3 translocates to and co-localizes with damaged mitochondria. These findings agree with Zhou et al.,^32^ who suggested NLRP3 localizes to superoxide-concentrated domains. Recently, we demonstrated that a mitochondria-targeted derivative of apocynin, mito-apocynin, reduces neuroinflammation, and prevents DAergic neurodegeneration in an MPTP mouse model of PD.^9^ In the present study, mito-apocynin pre-treatment attenuated rotenone-induced and tebufenpyrad-induced superoxide generation, which in turn reduced secretion of IL-1β in primed microglia. Furthermore, reducing the superoxide generation reduces the translocation of NLRP3, indicating the probable upstream role of mitochondrial superoxide in NLRP3 inflammasome activation.

Although we observed in the Seahorse Mito Stress test that rotenone and tebufenpyrad could diminish mitochondrial activity in unprimed microglia, at other endpoints the effect of the pesticides in unprimed cells was not that dramatic. This could be due to the high sensitivity of the Seahorse assay compared to other tests. Another possible reason is that, unlike neuronal cells, microglial cells may compensate the bioenergy deficits from extra-mitochondrial energy sources. Future studies will address this issue. Sherer et al. ^35^ reported the loss of ATP in tebufenpyrad treated cells at 6 h. In this study, most of the functional assays were performed at the 2-h to 3-h time point. Also, rotenone activates the p-38 MAPK and NFκB pathways in microglial cells,^56^ which may lead to proliferation of microglia. Further research needs to be performed to delineate the molecular mechanism that makes glial cells more resistant to mitochondrial inhibitors.

Lysosomal dysfunction and dysregulation of autophagy have been linked to PD.^57^ Downregulation of lysosomal function may lead to accumulation of misfolded proteins like α-synuclein, which has been linked to PD pathology. Recent studies have shown that lysosomal dysfunction and destabilization can lead to inflammasome activation in macrophages in Gaucher disease models.^58,59^ Furthermore, a compromised lysosomal membrane leads to release of lysosomal cathepsins, which can induce NLRP3 inflammasome activation.^48^ Here, we demonstrate that prolonged exposure to both rotenone and tebufenpyrad induced lysosomal dysfunction in primed microglial cells, pointing out a probable role for lysosome dysfunction in regulating neuroinflammation.

Neuroinflammation and mitochondria-derived superoxide generation play key roles in degeneration of DAergic neurons in PD.^15,16,60,61^ Inflammasome activation in glial cells has been shown to contribute to disease pathology in AD^28,62^ and TBI, wherein treatment with anti-ASC antibody after TBI was beneficial in a mouse model.^63^ Furthermore, induction of IL-1β and IL-18 was linked to higher susceptibility and progression of multiple sclerosis.^25^ Here, we show that conditioned media from primed microglial cells treated with complex-1 inhibitors triggered DAergic neurotoxicity in both primary mesencephalic cultures and differentiated LUHMES cells, suggesting that NLRP3 inflammasome activation in glial cells can potentially lead to neurodegeneration (Fig. 6). Furthermore, MCC-950, the NLRP3 inflammasome inhibitor, reduced the loss of TH-positive neurite length, suggesting the inflammasome’s probable role in neurodegeneration. The mouse model of rotenone (30 mg/kg, gavaged daily for 28 days) exhibits motor deficits, selective loss of nigrostriatal DAergic neurons and increased α-synuclein in DAergic neurons.^64^ The rotenone mouse model was re-evaluated by Inden et al.^38^ who used two doses (30 and 100 mg/kg) of rotenone for 56 days. But 100 mg/kg of rotenone for 28 days did not cause any change in DAergic neurons. Hence, for this study, we utilized the rotenone model and were able to demonstrate activation of the NLRP3 inflammasome in vivo (Fig. 1f–k) during mitochondrial impairment. The major source of NLRP3 activation leading to inflammation in the brain are microglial cells.^65^ In this study, we have also shown that rotenone exposure leads to increased NLRP3 in microglial cells in rats, further solidifying the activation of NLRP3 in microglial cells in vivo.

In various neurodegenerative disorders including PD, the underlying mechanism behind sustained chronic inflammation has still not been deciphered. In this study, we propose a mechanism for regulating chronic inflammation in PD. All our findings collectively show that microglial mitochondria play an important role in regulating the NLRP3 inflammasome pathway in PD models (Fig. 6d). In this study, we show that LPS priming induces NLRP3 and pro-IL-1β production but not capase-1 cleavage or assembly of the inflammasome complex in microglia, which is consistent with the current literature on the NLRP3 inflammasome signaling pathway. Pesticide exposure leads to inflammasome assembly and secretion of IL-1β. Furthermore, we show that the superoxides generated by mitochondrial dysfunction leads to NLRP3 translocation. Reducing this superoxide generation lowers the translocation and inflammasome activation, but inhibiting the activation of NLRP3 does not reduce the mitochondrial damage, indicating that mitochondrial dysfunction is upstream of inflammasome formation. Additionally, the inflammatory cascade is intensified by lysosomal dysfunction, leading to the accumulation of more damaged mitochondria and thus greater superoxide generation. Collectively, our study points to the existence of a complex interplay between microglial mitochondrial impairment and NLRP3 inflammasome signaling and its role in sustained neuroinflammation in DAergic neurodegenerative processes in PD.

## Materials and methods

### Chemicals and reagents

Rotenone (95–98% purity), oligomycin, FCCP and antimycin were purchased from Sigma, and tebufenpyrad (96% purity) was purchased from AK Scientific Inc. Dimethyl sulfoxide (DMSO) was purchased from Fisher Scientific. We purchased DMEM-F12, fetal bovine serum (FBS), L-glutamine, penicillin, streptomycin, MitoTracker Red, LysoTracker Green, and MitoSox Red stains from Invitrogen. The CellTiter 96 AQueous Non-Radioactive Cell Proliferation Assay kit and CellTiter Glo Luminescent Cell Viability Assay kit were obtained from Promega. ASC, NLRP3, and caspase-1 antibodies were purchased from Adipogen, and the TH antibody was obtained from Millipore. The CD11b magnetic separation kit was purchased from STEMCELL Technologies. LC3, AIM2, and NLRC4 antibodies were purchased from Cell Signaling Technologies. IL-1β antibody was purchased from R&D Technologies, while LAMP2 antibody was from Santa Cruz Biotechnology. Acetylated α-tubulin antibody and Duolink PLA red were obtained from Sigma. The MAP2 and Tuj1 antibodies and cathepsin D activity kit were obtained from Abcam. All the standards used for Luminex assay were purchased from PeproTech Inc. Streptavidin-biotin and biotinylated antibodies used for Luminex were purchased from eBioSciences. MCC-950 was obtained from Dr. Trent Woodruff’s lab, University of Queensland, Brisbane, Australia. Mito-Apocynin was obtained from Dr. Balaraman Kalyanaraman’s lab at the Medical College of Wisconsin in Milwaukee.

### Cell cultures and treatments

For primary microglial culture, one-day-old C57BL/6 pups were sacrificed, their brains dissected out, and a single cell suspension was prepared. After growing in culture for 16 days, the microglia were separated using a magnetic bead separation technique as previously described.^66,67^ Primary microglial cells were cultured in DMEM-F12, 10% FBS, 1% sodium pyruvate, 1% glutamine, 1% penicillin-streptomycin, and 1% non-essential amino acids. We also obtained a wild**-**type microglial cell line as a kind donation from Dr. D.T. Golenbock (University of Massachusetts Medical School, Worcester, MA). The wild-type microglial cell line was characterized by Halle et al.^28^ and cultured in DMEM medium, 10% FBS, 1% glutamine, and 1% penicillin-streptavidin. The DAergic Lund human mesencephalic (LUHMES) cell line was differentiated and maintained as described in our recent publication.^68^ Treatments were done in 2% FBS-containing medium. For LPS-priming treatments, cells were treated with LPS (1 µg/mL) for 3 h. Next, the cells were triple-washed with full serum medium to remove any LPS, and then mitochondrial complex-1 inhibitors were added to the cells (50 nM to 1 µM) for 2 to 24 h.

Primary mesensephalic neurons were isolated from gestational 15-day-old mouse embryos as described previously.^69^ These plated cells have a viability ranging from 70–80%. The primary cultures used were enriched in neurons. Briefly, mesencephalic tissues from E15 mouse embryos were dissected and maintained in ice-cold DMEM media and then dissociated in 10 mL of trypsin-0.25% EDTA (TE) for 15 min in a 37°C water bath with sporadic shaking. The action of TE was stopped using 20 mL DMEM containing 10% FBS. The trypsinized tissue was further washed twice in 10% DMEM followed by a final wash in neurobasal media. Tissue was titurated using a 10 mL sterile pipette and the resulting suspension was passed through a 70-micron filter. The dissociated cells were then plated at an equal density of 0.1 million cells per well on 12-mm coverslips precoated with 0.1 mg/ml poly-D-lysine. Cultures were maintained in neurobasal media fortified with B-27 supplement, 500 mM L-glutamine, 100 IU/mL penicillin, and 100 μg/mL streptomycin. The cells were maintained in a humidified CO2 incubator (5% CO2 and 37 °C) for 24 h. One-half of the culture media was replaced every other day, and 6- to 7-day-old cultures were used for experiments. These cells were maintained in neurobasal medium which selectively inhibits glial proliferation. Cells were treated with conditioned medium from primed and unprimed microglial cells for 24 h. For neurite length analysis, NeuronJ plugin in ImageJ was utilized. Only neurites in contact with the cell body were measured; neurites without cell body contacts were not measured.

### Animal studies

Eight-week-old male C57BL/6N mice (3–4 animals per group), obtained from Charles River, were housed under standard conditions: constant temperature (22 ± 1°C), humidity (relative, 30%), and a 12-h light/dark cycle. From the in vitro data, we estimated 3–4 animals per group as sufficient to detect significant biochemical changes. All mice were pre-screened for normal baseline performance during behavioral assessments conducted before randomly assigning animals to experimental groups. Investigators involved with data collection and analysis were not blinded to group allocation of mice. After acclimating for 3 days, mice were gavaged daily with 30 mg/kg of rotenone for 28 days, after which the mice were sacrificed. This protocol for chronic administration of rotenone has been reported to cause a significant loss of nigrostriatal DAergic neurons and behavioral impairment in mice.^38,64^ Sixteeen rats, age 6–7 months old, were purchased from Hilltop Lab Animals (Scottdale, PA). These animals were administered either rotenone (2.8 mg/kg/day, *n* = 8) or vehicle (*n* = 8) for 4 consecutive days via intraperitoneal (i.p) injection. The rotenone solution was first prepared as a 50 × stock in 100% DMSO and diluted in the medium-chain triglyceride Miglyol 812 N. Vortexing the solution created a stable emulsion with a final concentration of 2.8 mg/mL rotenone in 98% Miglyol 812 N and 2% DMSO. After the final injection, the animals were housed for 3 months. The animals were randomly assigned to treatment using an online randomization tool (random.org). Experimenters were blinded to each rat’s treatment allocation.

Use of the animals and protocol procedures were approved by the Institutional Animal Care and Use Committee (IACUC) at Iowa State University (Ames, IA, USA) and all methods were performed in accordance with relevant guidelines and regulations.

### Western blotting

Following the treatment of rotenone or tebufenpyrad for 3–6 h, primed or unprimed microglial cell pellets were lysed using modified RIPA buffer and sonicated. The proteins were normalized and 20–30 µg of protein was loaded in each lane and separated using a 12–15% SDS polyacrylamide gel as discussed previously.^70,71^ After transferring, nitro-cellulose membranes were blocked with LI-COR blocking buffer and washed with PBS mixed with 0.05% Tween. The membranes were then treated with the indicated primary antibodies, followed by incubation with IR-680 anti-mouse and IR-800 anti-rabbit secondary antibodies. The membranes were scanned using the Odyssey LI-COR Imaging System. The primary antibodies used for immunoblotting were NLRP3 (1:1000) (AB_2490202), Caspase-1 p20 (1:1000) (AB_2490248), and IL-1β (1:1000) (AB_416684). β-actin was used as a loading control.

### Immunocytochemistry (ICC)

Cells were plated on PDL-coated coverslips. After treatment for 2–6 h with a pesticide, primed or unprimed cells were fixed in 4% paraformaldehyde for 30 min followed by blocking with 1.5% BSA, 0.05% Tween, and 0.5% Triton for 1 h. Primary antibodies were prepared in 1% BSA and incubated overnight at 4°C. The following primary antibodies were used: ASC (1:400, AB_2490440), NLRP3 (1:500, AB_2490202), TH (1: 2000, AB_2201528), TH (1:1000, AB_696697), MAP2 (1:1000, AB_448205), Tuj1 (1:1000, AB_444319), LC3 (1:400, AB_2137716), acetylated α-tubulin (1:400, AB_477585), SIRT2 (1:500, AB_1142864) and LAMP2 (1:400). After primary antibody incubation, each well was washed with PBS five times and then incubated with secondary antibody for 1 h. Cell nuclei were stained with Hoechst (1:5000) and mounted on slides using the Fluoromount mounting medium (Sigma). Slides were dried overnight and then imaged using a Nikon Eclipse C1 microscope. For co-localization, a 3D color plotter (Fiji image analysis software) was used to plot all the different colors seen in the RGB images. Briefly, RGB images were opened using Fiji and then analyzed using its 3D color plotter plug-in, which plots every colored pixel it picks up in the RGB image along x, y, and z axes representing red, green and blue, respectively. For co-localization between the green and red channel, it plots the pixels along the diagonal of the red and green axes to visualize the co-localization. Furthermore, the resulting intensity from merging the red and green signals can also be quantified in this analysis.

### Luminex assays

Cytokine levels were assessed via Luminex assays according to a protocol by Panicker et al.^8^ Briefly, primary microglial cells were treated in 96-well plates (Figs. 2, 4 and 6) or 6-well plates (Fig. 1) with 100–900 μL of 2% FBS-containing DMEM-F12 medium for 6–24 h. After treatment, 40 μL of treatment medium was collected and added to 40 μL of primary antibody conjugated to magnetic microspheres and incubated overnight at 4 °C in a clear-bottom, black 96-well plate. After incubation, each well was triple-washed using a magnetic washer and then incubated for 1 h with secondary antibodies. Lastly, samples were incubated for 30 min with streptavidin/phycoerythrin. A Bio-Plex reader was used to read the 96-well plates. A standard curve of all the cytokines was prepared using standard cytokines.

### q-RT-PCR

RNA was isolated using TRIzol extraction methods as previously described.^72^ Following treatment for 2 h with the pesticides, RNA concentration was measured using NanoDrop. One microgram of RNA was used to convert RNA into cDNA using the High Capacity cDNA Reverse Transcription Kit (Applied Biosystems #4368814) following the manufacturer’s protocol. For qPCR, 10 µL of SYBR Green Mastermix (Qiagen Cat #208056), 1–2 µL of primers, 6–7 µL of water and 1–2 µL of cDNA were used. The following genes were used for q-RT-PCR: NLRP3 (primer sequences: forward-TGCTCTTCACTGCTATCAAGCCCT, and reverse-ACAAGCCTTTGCTCCAGACCCTAT, synthesized at Iowa State University’s DNA Facility), IL-1β, IL-18, NLRC4, and AIM2 (QuantiTect Primers, Qiagen). The housekeeping gene 18 S rRNA (Qiagen Cat #PPM57735E) was used in all qPCR experiments. No-template controls and dissociation curves were run for all experiments to exclude cross-contamination.

### Live cell staining

Cells were stained following the manufacturer’s protocol using tetramethylrhodamine methyl ester perchlorate (TMRM) (2-h treatment), MitoSox Red (3- to 6-h treatment), MitoTracker Red (6-h treatment), and LysoTracker Green (6-h treatment), all obtained from Molecular Probes and used according to our previous publication.^21^ Briefly, cells were treated either on PDL-coated coverslips or in 96-well plates. Following treatment, cells were washed twice with HBSS and then the dye was added according to the dilution recommended by manufacturer. After staining, cells were washed and then either fixed for ICC or imaged directly. Following 2-h pesticide exposure for TMRM, non-fixed cells were imaged under the FLoid Cell Imaging system (Thermo Fisher Scientific). For MitoSox Red imaging, a Cytation 3 real-time imaging system (BioTek) was used to take hourly images. Changes in mitochondrial structure were quantified using the ImageJ plugin “mitochondrial morphology,” developed by Ruben K Dagda (2010). We selected parameters for quantifying damage to mitochondrial structure based on recent studies.^21,31,73^

### ATP assay

Cells were plated in opaque-walled 96-well plates and treated with the toxicants for 2 h. After treatment, 100 μL of CellTiter-Glo Reagent was added to each well containing 100 μL treatment media and incubated for 5 min. Readings were taken using a luminometer.

### MTS mitochondrial activity assay

Cells were plated in 96-well plates, and after treatment for 3–24 h, 10 μL of MTS dye (Promega) was added to each well and incubated for 1.5 h. After incubation, a plate reader was used to take excitation and background (subtracted) readings at 490 and 660 nm, respectively.

### Mitochondrial dynamics analysis

A Seahorse XFe24 Analyzer was used to measure mitochondrial oxygen consumption rates and extracellular acidification rates using the Mito Stress test following a previously published protocol.^31^ Primary mouse microglia were plated at 90,000 cells/well on a Seahorse 24-well plate. The calibration plate was hydrated overnight in a non-CO_2_ incubator. All treatments were done in serum-free medium. Cells were primed with LPS for 3 h followed by pesticide exposure for 3 h. For the Mito-Stress test, 0.75 μM oligomycin, 1 μM FCCP, and 0.5 μM rotenone/antimycin were used. Mito-Stress report generator was used for analysis.

### Duolink proximal ligation assay (PLA)

Duolink PLA assay was performed following the manufacturer’s protocol.^74^ Briefly, 15-k primary microglial cells were plated on PDL-coated coverslips in 96-well cell culture plates. Following treatment for 3 h, cells were washed and fixed using 4% poly-D-lysine, blocked with blocking buffer, and incubated in primary antibodies overnight. After primary antibody incubation, the Duolink in situ Detection Reagents Red (Sigma) was used according to manufacturer’s protocol. Confocal imaging was performed on these coverslips at the Iowa State University Microscope Facility using a Leica DMEIR2 confocal microscope with 63X oil objective. For *z*-stacking, each image consisted of 0.5-µm sections and 5 slices.

### Cathepsin D assay

One million primary microglial cells were plated in PDL-coated, 12-well cell culture plates. Following a 6-h treatment of rotenone or tebufenpyrad, cells were collected and the cathepsin activity assay was performed following manufacturer’s protocol (Abcam).

### Statistical analysis

All in vitro data were determined from three to eight biological replicates. GraphPad 5.0 was used for statistical analysis with *p* ≤ 0.05 considered statistically significant. Two-way ANOVA was used for comparing multiple groups. In most cases, Bonferroni post analysis was applied. For comparing 2 groups, Student’s *t*-test was used. Where the normality assumption was violated, we conducted nonparametric tests, however, in no case did the nonparametric results change the overall interpretation of parametric results.

### Data availability

Raw data supporting the results reported in this article are in the figure source data files available upon request.

## Electronic supplementary material

Supplementary Information

Supplementary Video 1

Supplementary Video 2

Supplementary Video 3
