# Supplementary material for: Mitochondrial impairment in microglia amplifies NLRP3 inflammasome proinflammatory signaling in cell culture and animal models of Parkinson’s disease
Source: NPJ Parkinsons Dis. 2017 Oct 17;3:30. doi: 10.1038/s41531-017-0032-2 (PMC5645400; doi:10.1038/s41531-017-0032-2)
Supplement: Supplementary file 1 — Supplementary Information [file 41531_2017_32_MOESM1_ESM.docx]

Supplementary Materials


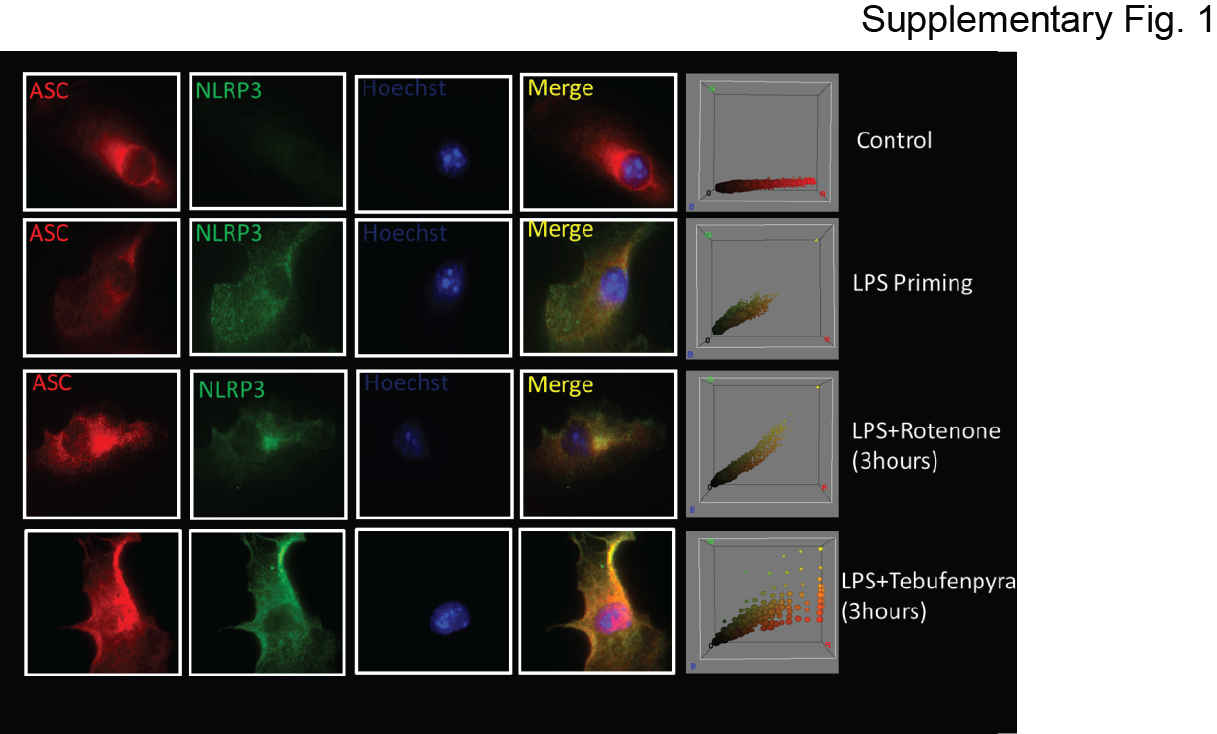


Supplementary Fig. 1: **LPS priming leads to NLRP3 upregulation in primary microglial cells.** ICC showing the upregulation of NLRP3 and co-localization with ASC after treatment with rotenone or tebufenpyrad for 2 h. The right-most panel is the representative 3D color plot. Scale bar, 20 μm.

Supplement
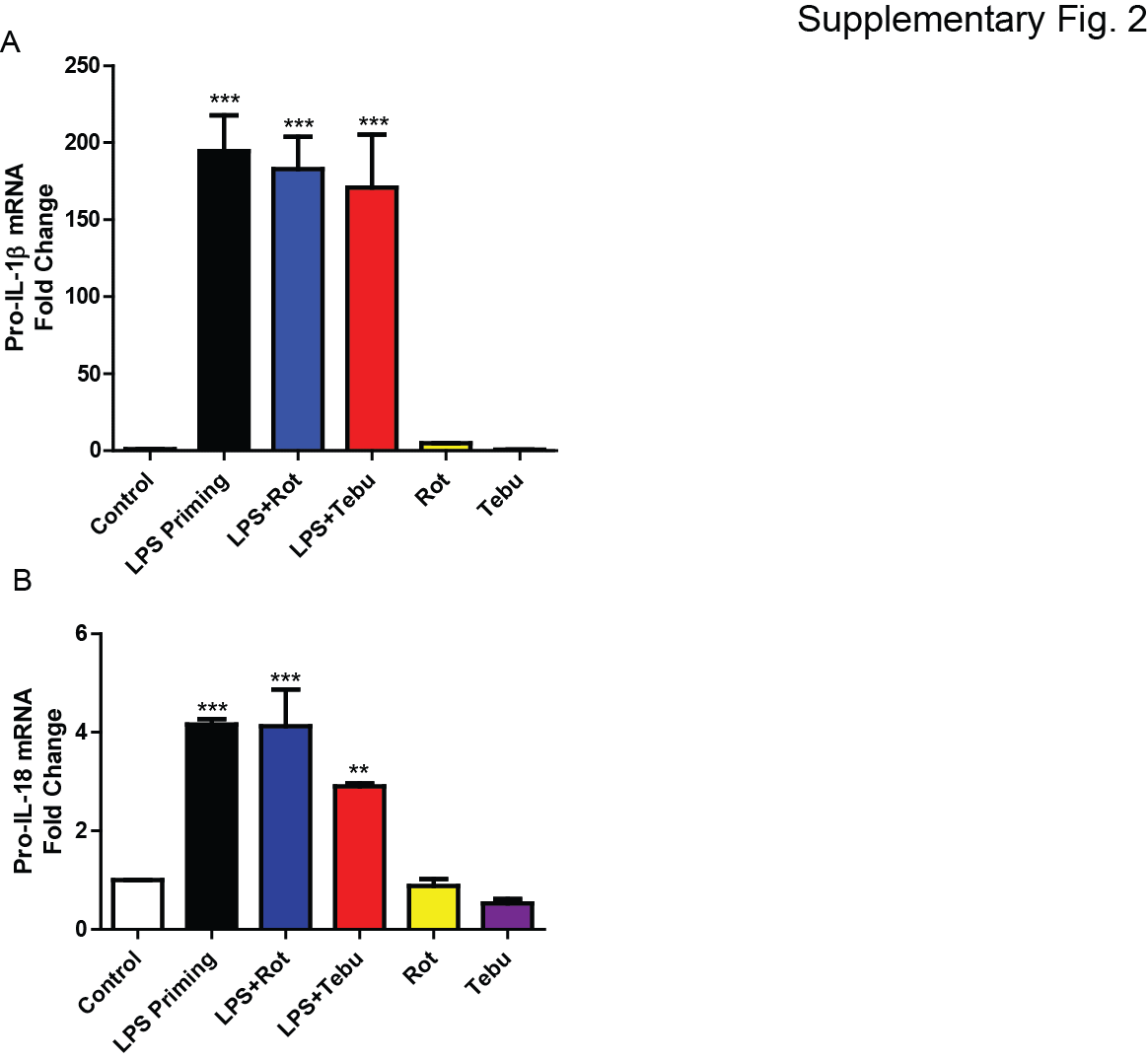
ary Fig 2: **Pesticide-induced inflammasome activation induces IL-1**β **and pro-IL-18.** q-RT-PCR analysis showing the gene levels of pro-IL-1β and pro-IL-18 in LPS-primed primary mouse microglia treated with or without 1 µM rotenone or tebufenpyrad for 2 h. Data analyzed via two-way ANOVA with Bonferroni adjustment, *p<0.05, **p<0.01, ***p<0.001 and are represented as Mean±SEM with n=3-8.


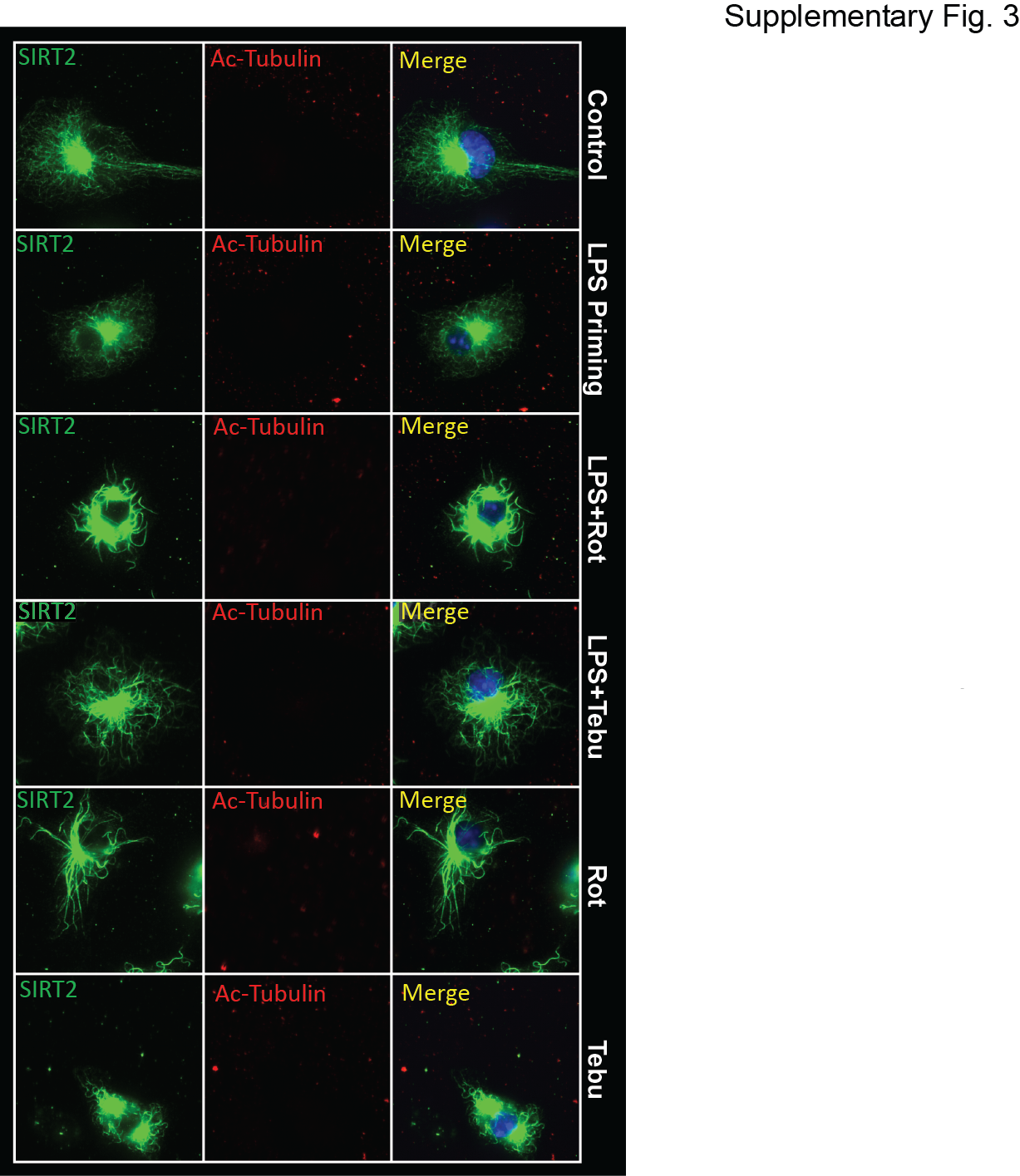


Supplementary Fig. 3: **Pesticide exposure does not induce acetylation of microtubules at early time points in microglial cells.** ICC analysis reveals that SIRT2 levels or acetylated α-tubulin did not significantly change after 2 h of 1-µM pesticide exposure in primed and unprimed primary microglial cells. Scale bar, 20 μm.


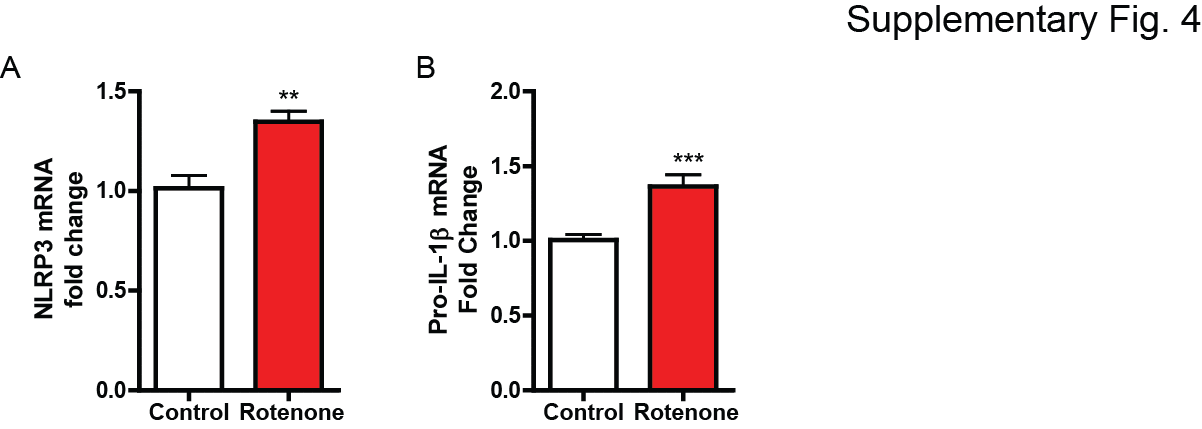


Supplementary Fig. 4: **Pesticide exposure alone induced NLRP3 and pro-IL-β in presence of neurons.** (A-B) qRT-PCR analysis showing gene level of NLRP3 (A) and pro-IL-1β (B) following 1 µM rotenone exposure for 6 h in presence of neurons. Data analyzed via two-way ANOVA with Bonferroni adjustment, *p<0.05, **p<0.01, ***<0.001 and are represented as Mean±SEM with n=3-8.


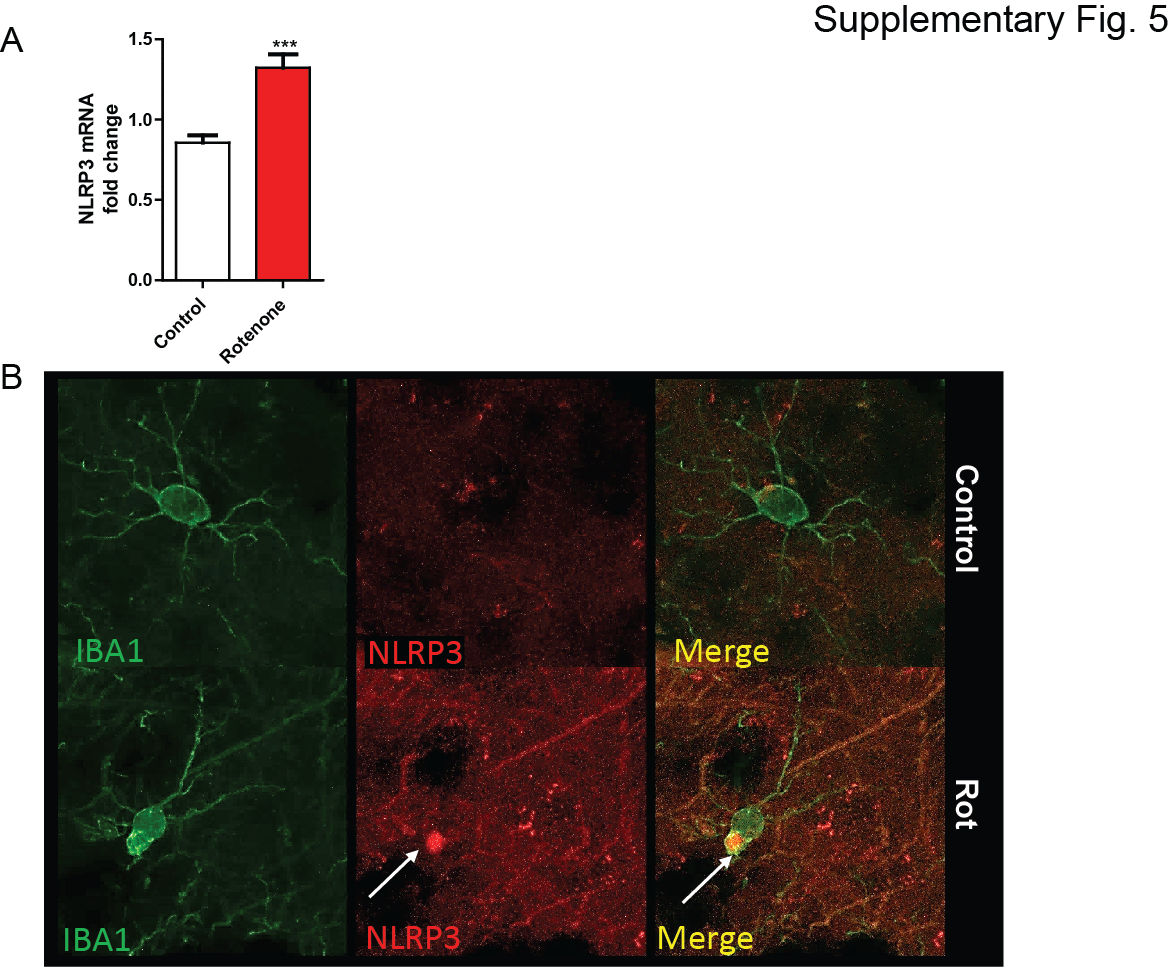
Supplementary Fig. 5: **Rotenone induced NLRP3 inflammasome activation in rat model.** (A) qRT-PCR analysis revealed NLRP3 upregulation in rotenone-treated rats. (B) IHC analysis showing increased NLRP3 in IBA1-positive cells in the striatum of rotenone treated rats. Data analyzed via Student’s t-test, *p<0.05, **p<0.01, ***<0.001 and are represented as Mean±SEM with n=4. Scale bar, 15 μm.


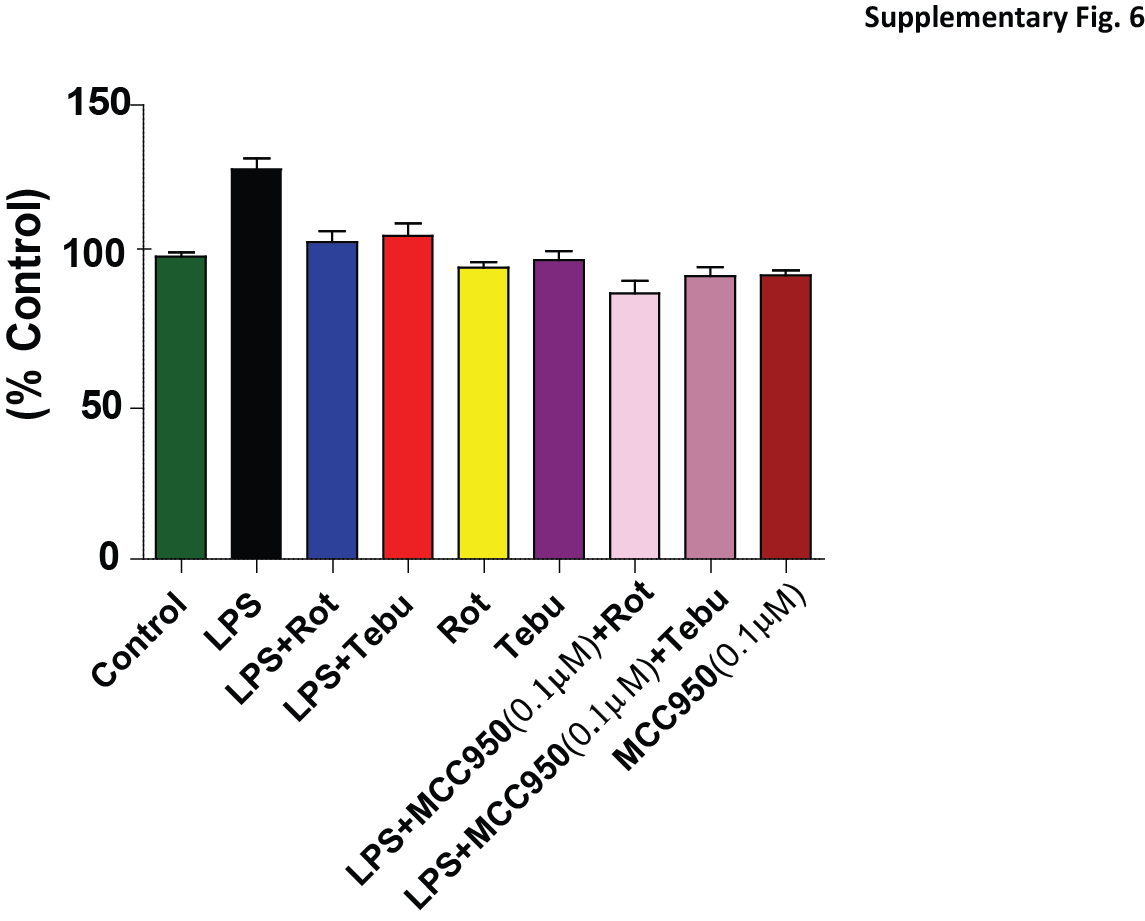
Supplementary Fig. 6:  **MCC950, an NLRP3-specific inhibitor, does not affect cell viability in primary microglial culture**. MTS assay of cell viability after treatment with MCC950. Data analyzed via two-way ANOVA with Bonferroni adjustment, *p<0.05, **p<0.01, ***<0.001 and are represented as Mean±SEM with n=3-8.


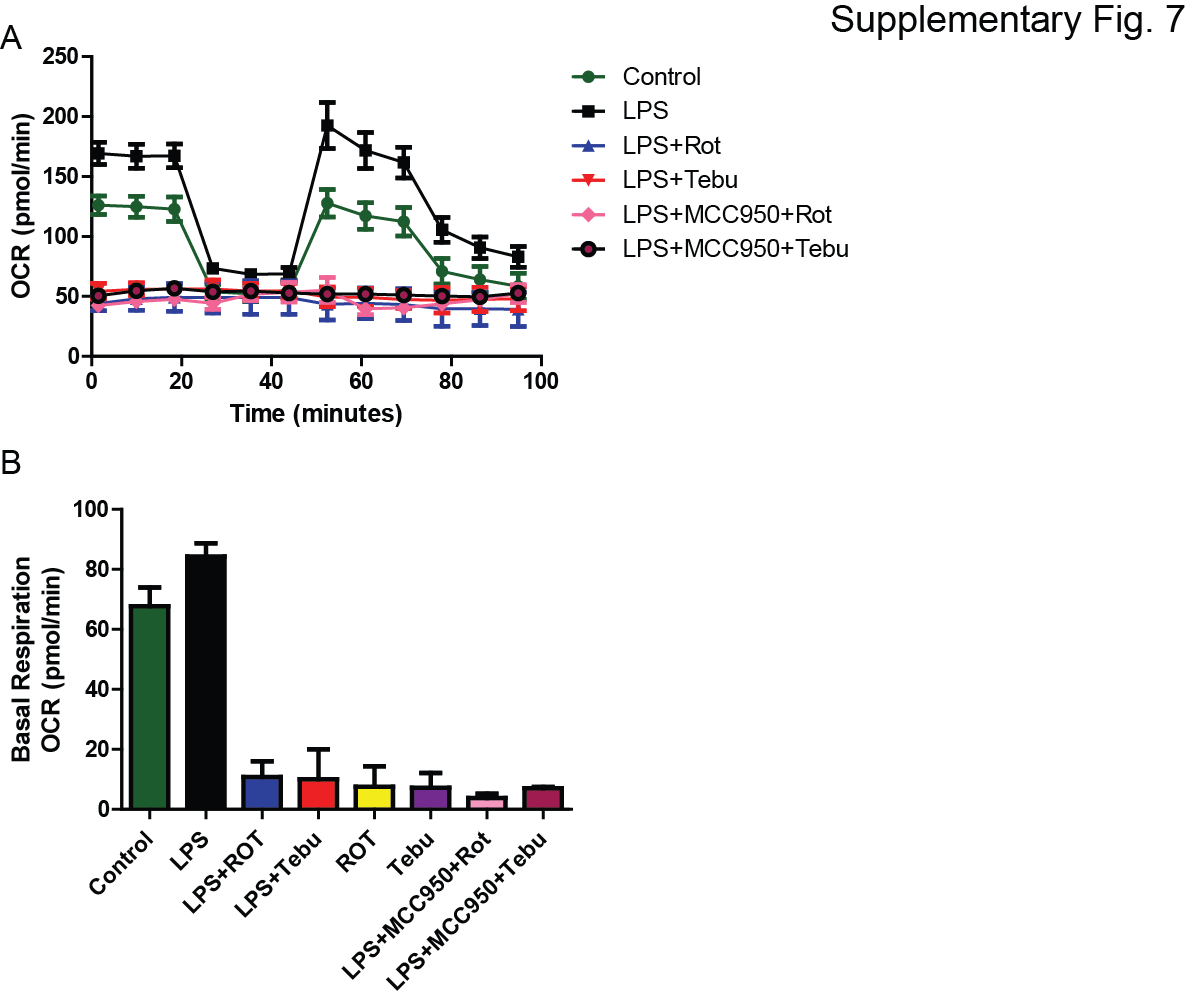


Supplementary Fig. 7: **MCC950 does not alter mitochondrial dynamics.** (A-B) Seahorse Mito Stress assays showing MCC-950 does not alter impaired mitochondrial bioenergetics in LPS-primed primary microglial cells treated with pesticides for 3 h. Data analyzed via two-way ANOVA with Bonferroni adjustment, *p<0.05, **p<0.01, ***<0.001 and are represented as Mean±SEM with n=3-8.


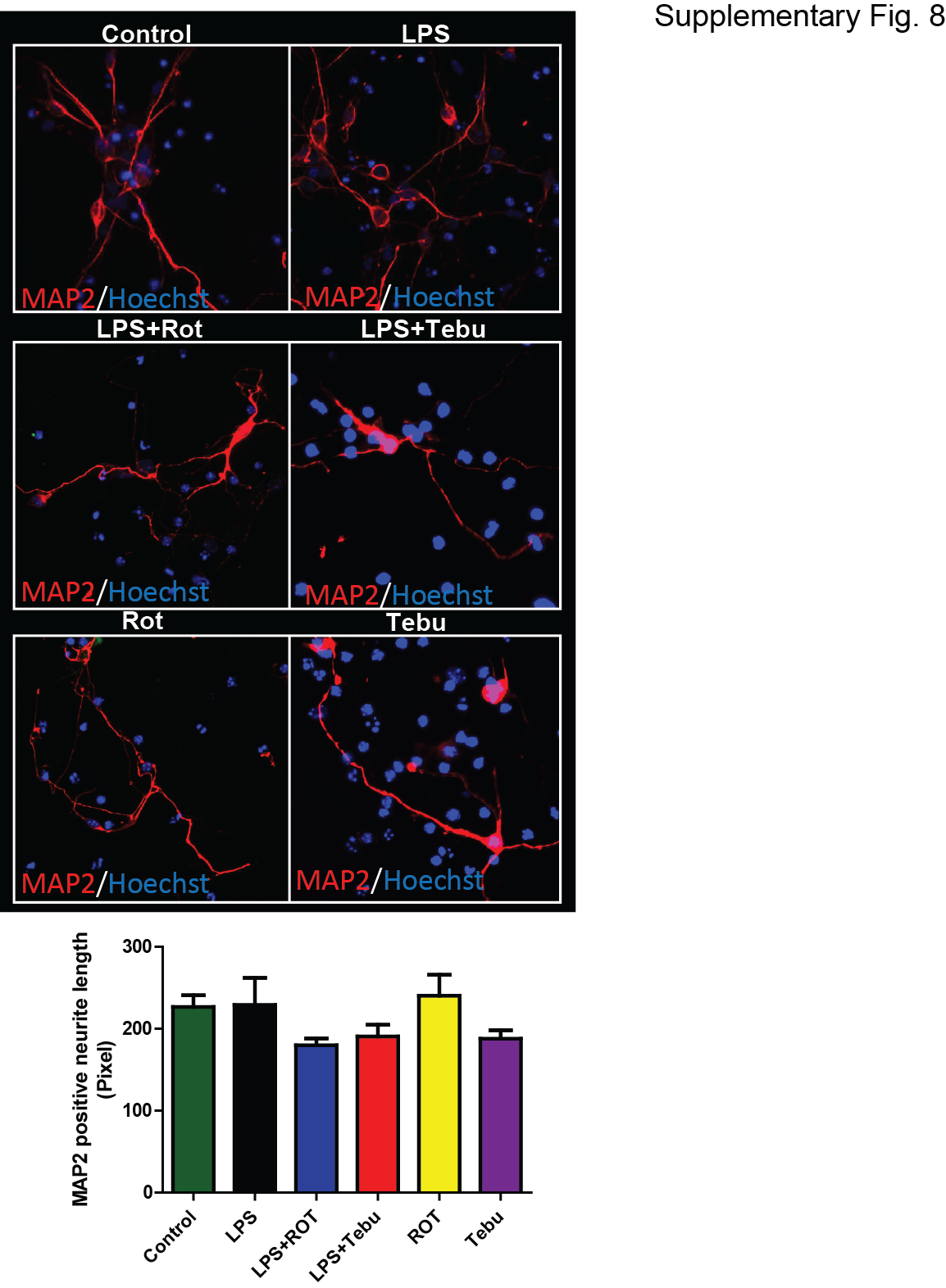


Supplementary Fig. 8: **Conditioned medium from pesticide-exposed microglial cells does not alter TH-negative neurite length.** ICC analysis showing that TH-negative neurite length is not affected on exposure to conditioned medium from primed microglial cells exposed to rotenone or pesticides. Data analyzed via two-way ANOVA with Bonferroni adjustment, *p<0.05, **p<0.01, ***<0.001 and are represented as Mean±SEM with n=3-8. Scale bar, 100 μm.


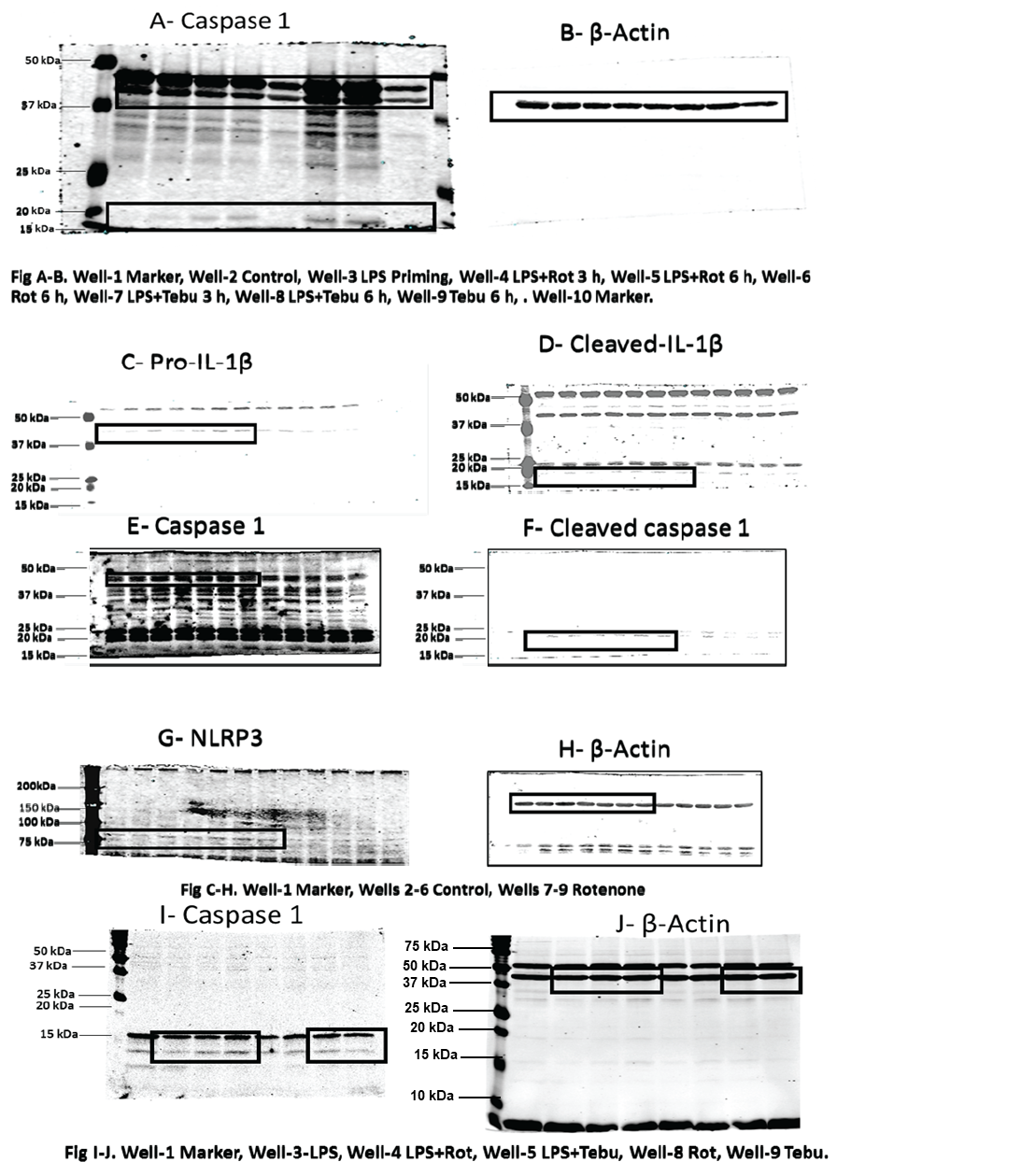


Supplementary Fig 9: **Full length Western blots shown in Fig 1.**  (A-B) Full length Western blots corresponding to Fig. 1C. (C-H) Full length Western blots corresponding to Fig. 1G. (I-J) Full Western blots corresponding to Fig. 5D. Samples derive from the same experiment and gels/blots were processed in parallel.

Supplementary Videos 1-3: **Time-lapse videos of mitochondrial superoxide generation in primed microglial cells treated with vehicle, rotenone, and tebufenpyrad, respectively.**
